# Obesity management in polycystic ovary syndrome: disparity in knowledge between obstetrician-gynecologists and reproductive endocrinologists in China

**DOI:** 10.1186/s12902-021-00848-w

**Published:** 2021-09-06

**Authors:** Ruilin Ma, Ying Zou, Wei Wang, Qingmei Zheng, Ying Feng, Han Dong, Zhangyun Tan, Xiaoqin Zeng, Yinqing Zhao, Yan Deng, Yanfang Wang, Aijun Sun

**Affiliations:** 1 Department of Obstetrics and Gynecology, Peking Union Medical College Hospital, Chinese Academy of Medical Sciences and Peking Union Medical College, Beijing, 100730 China; 2 Department of Obstetrics and Gynecology, Hunan Provincial Maternal and Child Health Care Hospital, Changsha, 410008 Hunan China; 3 Department of Reproductive Medicine, The Second Hospital of Hebei Medical University, Shijiazhuang, 050000 Hebei China; 4 Department of Gynecology, The Affiliated Hospital of Qingdao University, Qingdao, 266500 Shandong China; 5 Department of Obstetrics and Gynecology, The Second Affiliated Hospital of Nanchang University, Nanchang, 330006 Jiangxi China; 6 Department of Obstetrics and Gynecology, Women and Children’s Hospital of Jinzhou, Jinzhou, 121000 Liaoning China; 7 Department of Obstetrics and Gynecology, Xinhui Maternity and Children’s Hospital, Nanning, 529100 Guangxi China; 8 Department of Gynecology, Guangzhou Women and Children’s Medical Center, Guangzhou, 510000 Guangdong China; 9 Department of Obstetrics and Gynecology, Peking Union Medical College Hospital, No. 1 Shuaifuyuan, Dongcheng District, Beijing, 100010 China

**Keywords:** Obesity, Polycystic ovary syndrome, Oral glucose tolerance test, Lipid profile, Metformin

## Abstract

**Background:**

Obesity is associated with the development of polycystic ovary syndrome (PCOS) and contributes substantially to metabolic abnormalities in women with PCOS. The study aimed to describe and compare the practices of physicians in the diagnosis, evaluation, and treatment of obesity in patients with PCOS.

**Methods:**

Reproductive endocrinologists (Repro-Endo) and obstetrician-gynecologists (non-reproductive medicine specialty, OB-Gyn) in China participated in a survey, and their responses were analyzed using χ^2^ tests, Fisher exact tests, and multivariable logistic regression analysis.

**Results:**

The study analyzed 1318 survey responses (85.8% OB-Gyn; 97.3% women). Body mass index was the most common diagnostic criterion for obesity; only 1.3% of participants measured waist circumference to identify abdominal obesity. More Repro-Endo participants (25% of all participants) enquired about the psychological problems of patients with obesity than OB-Gyn participants, and 42.5% of participants reported ordering both a lipid profile and oral glucose tolerance test (OGTT) for patients with obesity and PCOS. Multivariable analysis, that included physician’s specialty, age, hospital grade, and number of patients with PCOS seen annually, revealed that OB-Gyn participants were less likely to order OGTT (OR, 0.3; 95% CI, 0.2–0.4) and lipid profile (OR, 0.2; 95% CI, 0.1–0.3) than Repro-Endo participants. The most common treatments for patients with PCOS were lifestyle modification (> 95%) and metformin (> 80%). More Repro-Endo participants prescribed metformin at a dose of 1.5 g/day compared with OB-Gyn (47.6% vs. 26.3%), and more OB-Gyn participants reported being unclear about the appropriate dosage of metformin for patients with obesity and PCOS (8.9% vs. 1.6%).

**Conclusion:**

Our survey identified knowledge gaps in metabolic screening for patients with obesity and PCOS and a disparity in the evaluation and treatment of obesity in PCOS among different specialties. Similarly, it highlights the need to improve obesity management education for physicians caring for women with PCOS.

**Supplementary Information:**

The online version contains supplementary material available at 10.1186/s12902-021-00848-w.

## Background

According to the results of the China Hypertension Survey (CHS), a nationally representative cross-sectional study among residents aged ≥18 years that was conducted from October 2012 to December 2015, more than two-fifths of Chinese adults were obese or overweight [1]. A high body mass index (BMI) and an unhealthy diet are the main risk factors for chronic diseases among Chinese residents [2]. Both being overweight and obese are associated with multiple comorbidities, including metabolic syndrome, type 2 diabetes mellitus (T2DM), cancer, cardiovascular diseases, and obstructive sleep apnea, and these may lead to mortality.

Polycystic ovary syndrome (PCOS) is a well-known endocrine/metabolic disorder that affects females of different age groups, including the age of puberty, childbearing, and perimenopause. Although the pathogenesis of PCOS remains unclear, the relationship between heredity and environmental factors may play a significant role in its development [3]. As one of the environmental factors, obesity, caused by unbalanced food intake and expenditure, and exposure to environmental chemicals disrupting endocrine functions during critical growth stages, contributes substantially to endocrine and metabolic disorders in patients with PCOS [4]. Approximately 37% of women with PCOS are obese or overweight in China [5]; furthermore, women with PCOS have more visceral fat than age-matched and BMI-matched controls [6]. Obesity, especially abdominal obesity, has been reported to aggravate insulin resistance (IR), hyperandrogenism, and dyslipidemia, and increase the risk for T2DM, impaired glucose tolerance (IGT), cardiovascular diseases, non-alcoholic fatty liver disease, and psychological problems in women with PCOS [6–8]. Therefore, prompt recognition and management of obesity and related metabolic complications in women with PCOS are crucial. The Chinese Medical Association, American College of Obstetricians and Gynecologists (ACOG), and international evidence-based guidelines have suggested that all patients with PCOS should be assessed for diabetes and cardiovascular risk factors, including BMI, waist circumference, oral glucose tolerance test (OGTT), and lipid profile, and lifestyle modification should be recommended in all those with PCOS to enhance the quality of life and modify risks for cardiovascular disease and diabetes [9–11].

Several international online surveys have focused on practices regarding the diagnosis and management of PCOS among physicians [12–14]. However, data on the management of obesity and metabolic screening in patients with PCOS are scarce. In addition, previous physician surveys showed differences in practice patterns between gynecologists and reproductive endocrinologists in the United States [14], as well as between gynecologists and medical endocrinologists in Australia [15]. Therefore, we surveyed reproductive endocrinologists (Repro-Endo) and obstetrician-gynecologists (non-reproductive medicine specialty, OB-Gyn) on their usual practice regarding the diagnosis, evaluation, and treatment of obesity in patients with PCOS. Our study aimed to promote evidence-based care, patient satisfaction, and minimize long-term morbidities in women with PCOS.

## Methods

The study was approved by the Ethics Committee of Peking Union Medical College Hospital, Chinese Academy of Medical Sciences (No. S-K1373).

### Study design, setting, and participants

The study was a nationwide, online survey conducted from September 1, 2020 to September 30, 2020. Physicians registered in online chat groups of the China Maternal and Child Health Association received an invitation to complete this questionnaire. Information about the questionnaire and the URL of the online survey was contained in the message. Participation was voluntary and anonymous. Completion of the survey was taken as consent to participate.

### Questionnaire content

The questionnaire included the following data: provider’s demographics, clinical specialty, hospital grade, practice characteristics, diagnostic criteria used for obesity, provider’s knowledge of evaluation and treatment of women with obesity and PCOS, and practices regarding weight loss in women with obesity and PCOS. The questionnaire was piloted with 30 obstetricians and gynecologists, and their feedback was incorporated; there was no ambiguity or doubt regarding the contents of the questionnaire. The full questionnaire details are provided in Supplementary material.

### Statistical analysis

Statistical analysis was performed using SPSS version 26.0 software (IBM, Armonk, NY, USA). Categorical data are presented as frequencies and percentages, and groups were compared using Pearson’s χ^2^ tests or Fisher’s exact tests when appropriate. Variables included in the multivariable logistic regression analysis were as follows: physician’s specialty, age, hospital grade, number of patients with PCOS seen annually, and proportion of patients with obesity and PCOS. A *p*-value of < 0.05 was considered statistically significant.

## Results

In total, 1415 questionnaires were completed and submitted. Questionnaires filled by non-obstetricians and gynecologists (*n* = 97) were excluded, and 1318 questionnaires were included in the final analysis.

### General characteristics of the participants

Table 1 summarizes the general characteristics of the 1318 participants, including 14.2% Repro-Endo and 85.8% OB-Gyn participants. Most participants were female and above 35 years of age. About 60% of the participants were from tertiary hospitals, and 34.6% were from secondary hospitals. More OB-Gyn participants saw fewer than 50 patients with PCOS per year, and more Repro-Endo participants saw 50–200 patients or more than 200 patients with PCOS per year. Most participants in the OB-Gyn group (71.6%) believed that the prevalence of overweight/obesity was less than 50% in patients with PCOS, while 80.7% of participants in the Repro-Endo group believed that the prevalence of overweight/obesity was 50–80%. Menstrual disorder (80.6%) was the most common reason for patient’s visit, followed by infertility (17.1%). Participants in the OB-Gyn group saw more patients with menstrual disorders, and the Repro-Endo participants saw more patients with infertility. A few patients with PCOS (1.6%) consulted a doctor primarily for obesity or IR, and fewer patients with PCOS saw a doctor primarily for hirsutism or acne (Table 1).

**Table 1:** General characteristics of 1318 respondents

	Overall (***n*** = 1318)	Repro-Endo (***n*** = 187)	OB-Gyn (***n*** = 1131)	***P***-value
**Sex**				
female	1282 (97.3)	177 (94.7)	1105 (97.7)	.018
male	36 (2.7)	10 (5.3)	26 (2.3)	.018
**Age**				
18–35	226 (17.1)	22 (11.7)	204 (18.0)	.035
36–45	514 (39.0)	76 (40.7)	438 (38.7)	NS
≥ 46	578 (43.9)	89 (47.6)	489 (43.3)	NS
**Hospital grade**				
3	793 (60.2)	123 (65.8)	670 (59.2)	NS
2	456 (34.6)	61 (32.6)	395 (34.9)	NS
1	69 (5.2)	3 (1.6)	66 (5.9)	.016
**No. of patients with PCOS treated annually**				
< 50	849 (64.4)	54 (28.8)	795 (70.3)	<.001
51–200	358 (27.2)	86 (46.0)	272 (24.0)	<.001
> 200	111 (8.4)	47 (25.2)	64 (5.7)	<.001
**Main reason for clinic attendance in patients with PCOS**				
Menstrual disorders	1062 (80.6)	130 (69.5)	932 (82.4)	<.001
Infertility	226 (17.1)	54 (29.0)	172 (15.2)	<.001
Hirsutism/acne	9 (0.7)	1 (0.5)	8 (0.7)	NS
Obesity/IR	21 (1.6)	2 (1.0)	19 (1.7)	NS
**Estimated national prevalence of overweight/obesity in patients with PCOS**				
0–30%	384 (29.1)	28 (15.0)	356 (31.5)	<.001
31–50%	541 (41.1)	87 (46.5)	454 (40.1)	NS
51–80%	331 (25.1)	64 (34.2)	267 (23.6)	.002
> 80%	62 (4.7)	8 (4.3)	54 (4.8)	NS

*Abbreviations*: *Repro-Endo* Reproductive endocrinologist, *OB-Gyn* Obstetrician-gynecologists, *PCOS* Polycystic ovary syndrome

### Assessment of women with obesity and PCOS

BMI was the most common diagnostic criterion used for obesity. Most participants (64.7% of Repro-Endo, 58.7% of OB-Gyn participants, *P* = 0.122) chose the Chinese criteria (BMI ≥24 kg/m^2^ for overweight and ≥ 28 kg/m^2^ for obesity) when classifying patients’ weight. Overall, only 1.3% of the participants preferred measuring waist circumference to determine abdominal obesity, including 2.7 and 1.1% of those in the Repro-Endo and OB-Gyn groups, respectively (*P* = 0.065) (Table 2).

**Table 2:** Practices of physicians in the management of patients with obesity and PCOS

	Overall (***n*** = 1318)	Repro-Endo (***n*** = 187)	OB-Gyn (***n*** = 1131)	***P***-value
**Commonly used diagnostic criterion for obesity**				
BMI (WHO criteria)	353 (26.7)	29 (15.5)	324 (28.6)	<.001
BMI (Chinese criteria)	785 (59.6)	121 (64.7)	664 (58.7)	NS
BMI (Asian criteria)	164 (12.4)	32 (17.1)	132 (11.7)	.037
Measure waistline	16 (1.3)	5 (2.7)	12 (1.1)	NS
**Inquiry about family history of diabetes**				
Yes	1261 (95.7)	180 (96.3)	1081 (95.6)	NS
**Inquiry about family history of cardiovascular disease**				
Yes	882 (66.9)	131 (70.1)	751 (66.4)	NS
**Inquiry about family history of PCOS**				
Yes	1179 (89.5)	168 (89.8)	1011 (89.4)	NS
**Inquiry about symptoms of anxiety, depression, and other psychological problems**				
Yes	336 (25.5)	105 (56.1)	231 (20.4)	<.001

*Abbreviations*: *Repro-Endo* Reproductive endocrinologist, *OB-Gyn* Obstetrician-gynecologists, *PCOS* polycystic ovary syndrome, *BMI* Body mass index, *WHO* World Health Organization, *NS* No significant

Only 25.5% of the participants inquired about symptoms of anxiety, depression, and other psychological problems in patients with obesity and PCOS. This included 56.1 and 20.4% of participants in the Repro-Endo and OB-Gyn groups, respectively (*P* < 0.001; Table 2).

**Fig. 1:**
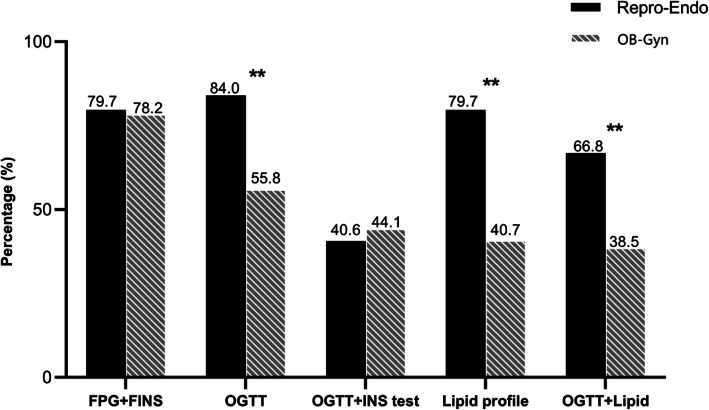
Metabolic screening tests used for patients with obesity and PCOS by Repro-Endo and OB-Gyn. Repro-Endo, reproductive endocrinologist; OB-Gyn, obstetrician-gynecologists; FPG, fasting plasma glucose; FINS, fasting insulin; OGTT, oral glucose tolerance test. *p < 0.05, **p < 0.001

The most frequently ordered metabolic screening tests for patients with obesity and PCOS among all the participants were fasting glucose and fasting insulin tests (78.5%), followed by OGTT (59.8%) and lipid profile (46.2%) (Fig. 1). About 2 in 5 participants (42.5%) reported ordering both lipid profile and OGTT for patients with obesity and PCOS.

In univariate analysis, physicians in secondary and tertiary hospitals, those who saw more than 50 patients with PCOS annually, and those who estimated that the prevalence of overweight/obesity was 51–80% were more likely to order OGTT for patients with obesity and PCOS. Physicians who saw 51–200 patients with PCOS annually and estimated the prevalence of overweight/obesity as 51–80% were more likely to order a lipid profile test for patients with obesity and PCOS (Table 3). Multivariate regression analysis revealed that participants in the OB-Gyn group were less likely to order an OGTT (odds ratio (OR) 0.3, 95% confidence interval (CI) 0.2–0.4) and a lipid profile test (OR 0.2; 95% CI 0.1–0.3) (Table 3) than those in the Repro-Endo group.

**Table 3:** Independent associations between physician characteristics and likelihood of physicians ordering OGTT and lipid profiles for patients with PCOS using multivariable logistic regression analysis

Variables	Physicians ordering OGTT	Physicians not ordering OGTT	Crude	Adjusted	Physicians ordering lipid profiles	Physicians not ordering lipid profiles	Crude OR	Adjusted OR
**Age**								
18-35	5 (0.6)	3 (0.6)	1	1	4 (0.7)	4 (0.6)	1	1
36-45	121 (15.4)	97 (18.3)	0.8 (0.2, 3.2)	0.8 (0.2, 3.3)	101 (16.5)	117 (16.5)	0.9 (0.2, 3.5)	0.9 (0.2, 3.8)
≥46	662 (84.0)	430 (81.1)	0.9 (0.2, 3.9)	0.9 (0.2, 3.8)	504 (82.8)	588 (82.9)	0.9 (0.2, 3.5)	0.8 (0.2, 3.4)
**Hospital grade**								
1	31 (3.9)	38 (7.2)	1	1	29 (4.8)	40 (5.6)	1	1
2	263 (33.4)	193 (36.4)	1.7 (1.0, 2.8) ^**a**^	1.4 (0.9, 2.4)	203 (33.3)	253 (35.7)	1.1 (0.7, 1.8)	0.9 (0.5, 1.6)
3	494 (62.7)	299 (56.4)	2.0 (1.2, 3.3) ^**a**^	1.7 (1.0, 2.8) ^**a**^	377 (61.9)	416 (58.7)	1.3 (0.8, 2.1)	1.0 (0.6, 1.7)
**Specialty**								
Repro-Endo	157 (19.9)	30 (5.7)	1	1	149 (24.5)	38 (5.4)	1	1
OB-Gyn	631 (80.1)	500 (94.3)	0.2 (0.2, 0.4) ^**a**^	0.3 (0.2, 0.4) ^**a**^	460 (75.5)	671 (94.6)	0.2 (0.1, 0.3) ^**a**^	0.2 (0.1, 0.3) ^**a**^
**Annual patients with PCOS**								
<50	469 (59.5)	380 (71.7)	1	1	364 (59.8)	485 (68.4)	1	1
51-200	242 (30.7)	116 (21.9)	1.7 (1.3, 2.2) ^**a**^	1.3 (1.0, 1.8)	188 (30.9)	170 (24.0)	1.5 (1.2, 1.9) ^**a**^	1.1 (0.9, 1.5)
>200	77 (9.8)	34 (6.4)	1.8 (1.2, 2.8) ^**a**^	1.2 (0.7, 1.9)	57 (9.3)	54 (7.6)	1.4 (0.9, 2.1)	0.7 (0.5, 1.2)
**Proportion of obesity in PCOS**								
0-30%	209 (26.5)	175 (33.0)	1	1	161 (26.4)	223 (31.5)	1	1
31-50%	321 (40.7)	220 (41.5)	1.2 (0.9, 1.6)	1.0 (0.8, 1.4)	248 (40.8)	293 (41.3)	1.2 (0.9, 1.5)	1.0 (0.8, 1.4)
51-80%	222 (28.2)	109 (20.6)	1.7 (1.3, 2.3) ^**a**^	1.4 (1.0, 1.9) ^**a**^	167 (27.4)	164 (23.1)	1.4 (1.1, 1.9) ^**a**^	1.2 (0.9, 1.6)
>80%	36 (4.6)	26 (4.9)	1.2 (0.7, 2.0)	1.1 (0.6, 1.9)	33 (5.4)	29 (4.1)	1.6 (0.9, 2.7)	1.6 (0.9, 2.7)

*Abbreviations*: *OGTT* Oral glucose tolerance test, *Repro-Endo* Reproductive endocrinologist, *OB-Gyn* Obstetrician-gynecologists, *PCOS* Polycystic ovary syndrome

^a^*P*< 0.05

### Management of women with obesity and PCOS

**Fig. 2:**
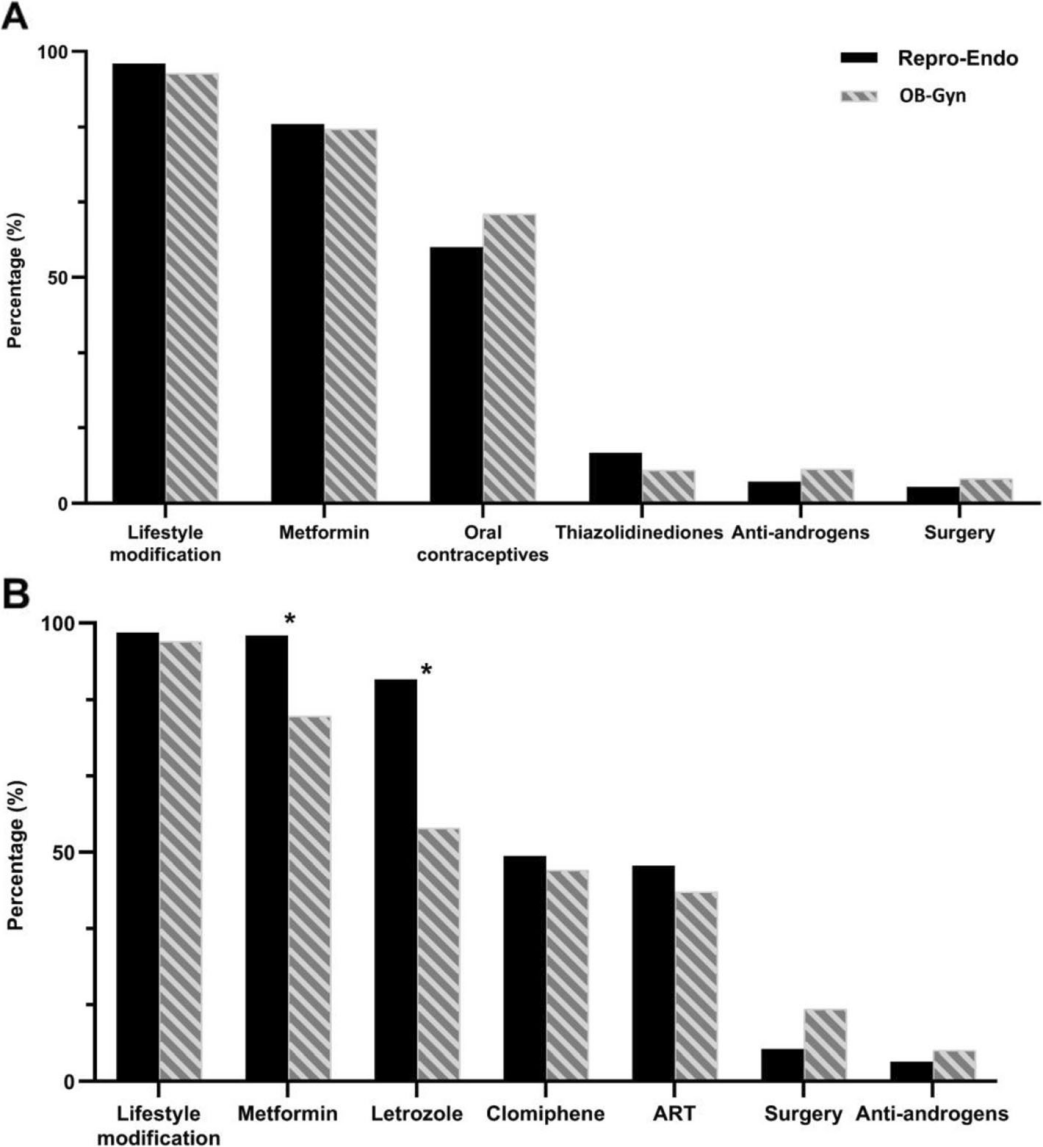
Treatment recommendations for women with obesity and PCOS. **a** Patients with no fertility demand. **b** Patients with fertility demand. Repro-Endo, reproductive endocrinologist; OB-Gyn, obstetrician-gynecologists; ART, assisted reproduction technology. *p < 0.05

When asked about the commonly prescribed treatments for patients with obesity and PCOS with or without fertility requirements, the participants’ first choice was lifestyle modification (> 95%), followed by metformin (> 80%) in both groups (Fig. 2). Participants in the Repro-Endo group were more likely to prescribe metformin for patients with obesity and fertility concerns (97.3% vs. 79.7%, *P* < 0.001) (Fig. 2b) than those in the OB-Gyn group.

**Fig. 3:**
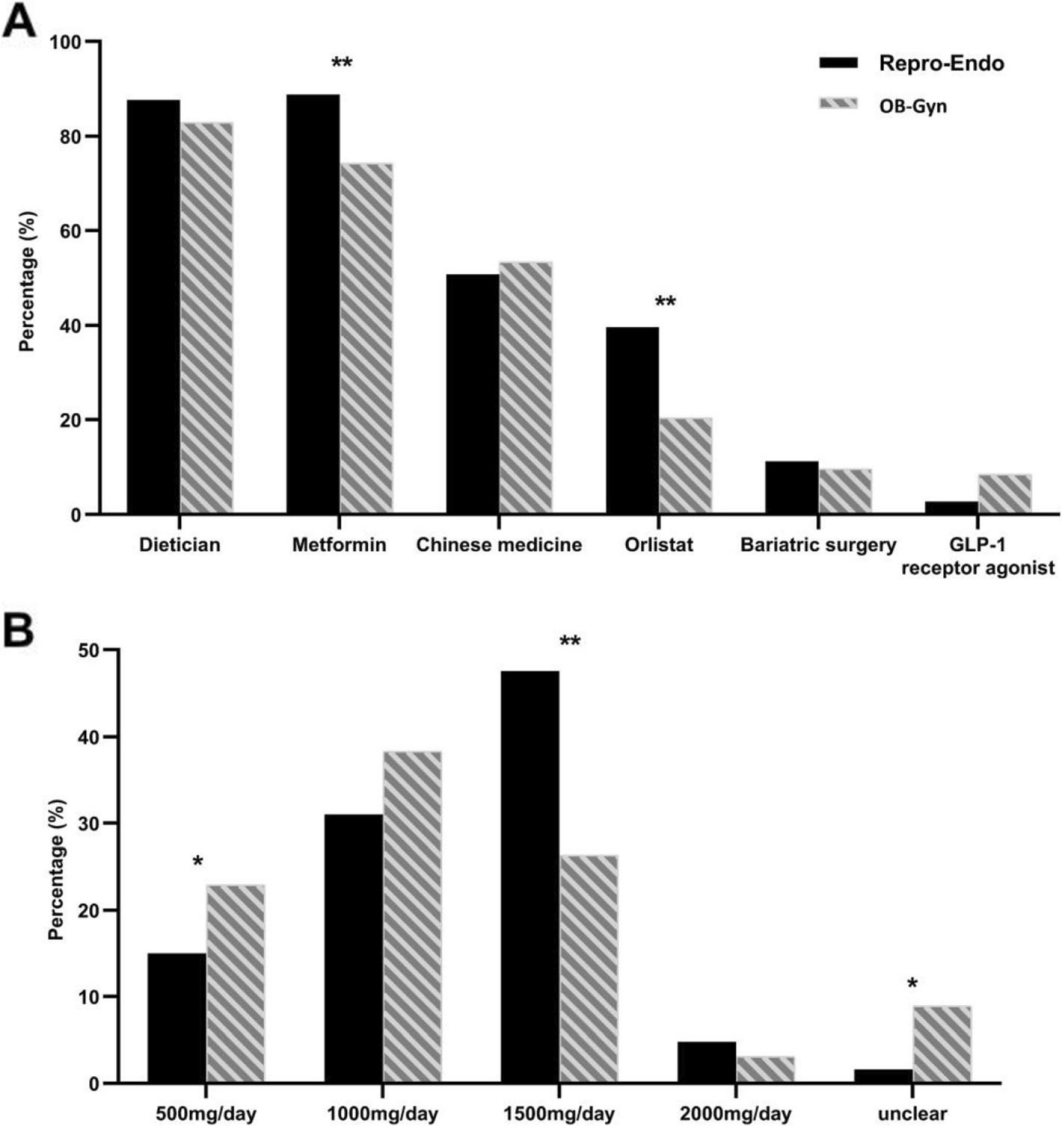
Suggestions for weight loss. **a** Recommended way to lose weight for women with obesity and PCOS. **b** Most common dosage of metformin prescribed in women with obesity and PCOS. Repro-Endo, reproductive endocrinologist; OB-Gyn, obstetrician-gynecologists; GLP-1 receptor agonist, glucagon-like peptide-1 receptor agonist. *p < 0.05, **p < 0.001

When physicians were asked how they would advise patients with obesity and PCOS to lose weight, the most common strategy in both groups was “referral to a clinical dietitian” (87.7% vs. 82.9%). In addition, more Repro-Endo participants prescribed metformin than those in the OB-Gyn group for patients with obesity desiring weight loss (88.8% vs. 74.3%, *P* < 0.001). The third suggestion for weight loss in both groups was Traditional Chinese medicine (TCM), including 50.8 and 53.4% of participants in the Repro-Endo and OB-Gyn groups, respectively (*P* = 0.509) (Fig. 3a). Overall, the most common dosage of metformin was 1000 mg/day (37.3%), followed by 1500 mg/day (29.3%). More Repro-Endo participants recommended metformin at a dosage of 1500 mg/day than OB-Gyn participants (47.6% vs. 26.3%, *P* < 0.001), whereas more OB-Gyn participants reported being unclear about the appropriate dosage of metformin for patients with obesity and PCOS than Repro-Endo participants (8.9% vs. 1.6%, *P* = 0.001) (Fig. 3b). Moreover, only 7.7% of the participants stated they would prescribe glucagon-like peptide-1 (GLP-1) receptor agonist for patients with obesity and PCOS for weight loss (Fig. 3a).

## Discussion

Obesity is a common feature of PCOS, and it aggravates reproductive and metabolic abnormalities in these patients. Among endocrinologists in Europe and OB-gynecologists and endocrinologists in America (ACOG and American Society for Reproductive Medicine (ASRM) members), obesity was the most important long-term concern of patients with PCOS [13, 14]. To the best of our knowledge, our study is the largest survey conducted to determine the practice and pattern of Chinese physicians in the assessment and management of obesity in PCOS patients. We observed that a significant percentage of physicians did not consider abdominal obesity and psychological problems in patients with PCOS. They did not routinely conduct OGTT and lipid profile in patients with obesity and PCOS, as recommended by current guidelines [9–11]. Most participants were aware of the benefits of lifestyle modification on obesity-related symptoms and recommended patients with obesity to consult a clinical dietitian. Moreover, metformin was widely used to improve insulin sensitivity and weight loss. Our results indicate significant differences between Repro-Endo and OB-Gyn specialists in their evaluation and treatment of patients with obesity and PCOS. Similarly, this highlights the need to educate physicians caring for women with obesity and PCOS.

Most participants in this study reported BMI as a diagnostic criterion for obesity. As height and weight are easily accessible anthropometric indicators, BMI is the most widely used measure of obesity worldwide. Nonetheless, studies have shown that metabolic dysfunction in patients with obesity may not be related to weight but to abnormal fat distribution, and abdominal obesity is an important risk factor for metabolic syndrome, diabetes, and cardiovascular diseases [16, 17]. The waistline is recognized as the most simple and practical indicator of abdominal adiposity; however, only a few participants in this survey preferred measuring the waistline of patients with obesity and PCOS.

A recent meta-analysis reported that women with PCOS were more likely to develop moderate and severe depression and anxiety symptoms. Although the etiology of this association remains unclear, these symptoms weakly correlated with age, BMI, elevated testosterone levels, and IR [8]. Moreover, obesity is associated with a decline in the quality of life of women with PCOS [18]. Our study showed that more Repro-Endo participants inquired about psychological problems of women with obesity and PCOS than OB-Gyn participants; however, these providers accounted for a small proportion of all participants. However, we did not survey physicians on their practices regarding psychological counseling.

Current guidelines recommend that two-hour OGTT and fasting lipid and lipoprotein levels should be measured in patients with obesity and PCOS at the time of diagnosis to evaluate metabolic abnormalities [9–11]. Obesity substantially contributes to metabolic abnormalities in women with PCOS, as it leads to dyslipidemia, IR, and IGT [19]. Abdominal obesity, dyslipidemia, and IGT are central components of metabolic syndrome and are notable risk factors of diabetes and cardiovascular disease [20, 21]. An online survey of ACOG members showed that metabolic screening in patients with PCOS was largely underutilized among OB-Gyn [22]. In this study, only 42.5% of participants reported ordering both a lipid profile and OGTT for patients with obesity and PCOS. Moreover, there were significant differences between the Repro-Endo and OB-Gyn participants in implementing guideline-recommended metabolic screening tests. Consequently, women with PCOS may be incompletely evaluated when seen by different specialists.

Nearly all participants reported lifestyle modification as the first-line treatment for patients with PCOS with or without fertility requirements, and most of them advised patients with obesity and PCOS to visit a clinical dietitian. This indicates a tendency toward a multidisciplinary strategy for obesity management. Metformin, an insulin sensitizer, is commonly used in combination with lifestyle modification to treat PCOS. Improvement in insulin sensitivity due to metformin administration is associated with its ability to decrease androgen levels, increase ovulation rate, and improve glucose tolerance [23]. Physicians in this survey generally accepted the weight-loss effect of metformin. Metformin can be safely administered at a dosage ranging from 500 to 2550 mg/day; however, its gastrointestinal side effects are dose-related. Therefore, the dosage of metformin is increased gradually. Patients with T2DM benefit most when taking metformin at the upper recommended daily dosage [24], and patients with IR and IGT taking metformin at 1500 mg/day benefitted from a reduction in fasting blood glucose and OGTT-2 h blood glucose levels and homeostatic model assessment of insulin resistance (HOMA-IR) index than those taking metformin at 500 mg/day [25]; however, a few studies have compared the efficacy of different doses of metformin on PCOS. A multicenter study reported that metformin administered at 1000 mg/day or 1500–1700 mg/day showed similar therapeutic effects on the clinical and biochemical parameters of PCOS patients [26]. Similarly, there was evidence that higher doses of metformin resulted in better weight loss in patients with obesity and PCOS [27]. Our results indicate a disparity in knowledge regarding the use of metformin between groups, which may lead to inadequate treatment for patients with obesity and PCOS.

More than half of the participants in this survey recommended TCM therapy for weight loss. TCM therapy, as an approach of complementary and alternative medical (CAM) treatment, has been used in women with PCOS to improve ovulatory dysfunction, IR, hyperandrogenism, and hyperlipemia either in combination with modern medicine or alone [28]. Moreover, the application of acupuncture or electroacupuncture for overweight/obese women with PCOS has been a common practice in Chinese medicine and has proven helpful for patients with weight loss [29]. TCM is a promising therapy for overweight/obese patients with PCOS, and high-quality trials are still needed to assess the efficacy of TCM for weight loss.

The GLP-1 receptor agonist is a new type of antidiabetic drug that has been used in the treatment of PCOS in recent years. GLP-1 receptor agonists have been shown to be effective in improving IR and IGT and are equally associated with weight loss due to delayed gastric emptying and increased satiety via a central action [30, 31]. Studies have shown that the GLP-1 receptor agonists are superior to metformin in reducing weight and improving IR [32]. Participants in this survey appeared to be less aware of GLP-1 receptor agonists. Nevertheless, more clinical trials and cost-benefit analyses are needed to definitively guide physicians in the use of the GLP-1 receptor agonist as a treatment option for PCOS.

## Conclusion

In conclusion, this survey appears to be the largest study to evaluate the patterns of practice regarding the management of obesity in PCOS patients across China. The results of this survey not only generate a clear picture of the present status of the management of obesity and PCOS, but also helps in identifying some underlying problems in practice. Nonetheless, there were some limitations to this study. First, the age and specialty of the participants were unevenly distributed. Second, only a small number of reproductive endocrinologists were noted. Finally, we did not survey physicians with respect to their practices regarding follow-up visits of patients with obesity and PCOS; additionally, we did not survey the age of patients with obesity and PCOS. Therefore, further investigations are needed to confirm and update our conclusions.

Our results identified knowledge gaps in metabolic screening for patients with obesity and PCOS as well as disparities in their evaluation and treatment among different specialties. Similarly, it highlights the potential for improvement in obesity management education for physicians caring for women with PCOS. Further research is needed to identify gaps in the care of patients with obesity and PCOS, improve the practice of physicians in the management of obesity and PCOS, and emphasize the importance of obesity prevention from childhood or adolescence.

## Supplementary Information

**Additional file 1.**

## Data Availability

The datasets used and/or analysed during the current study available from the corresponding author on reasonable request.

## Abbreviations

CHS: China Hypertension Survey
BMI: Body mass index
T2DM: Type 2 diabetes mellitus
PCOS: Polycystic ovary syndrome
IR: Insulin resistance
IGT: Impaired glucose tolerance
ACOG: American College of Obstetricians and Gynecologists
OGTT: Oral glucose tolerance test
Repro-Endo: Reproductive endocrinologists
OB-Gyn: Obstetrician-gynecologists
GLP-1 receptor agonist: Glucagon-like peptide-1 receptor agonist
ASRM: American Society for Reproductive Medicine
