# Supplementary material for: Obesity management in polycystic ovary syndrome: disparity in knowledge between obstetrician-gynecologists and reproductive endocrinologists in China
Source: BMC Endocr Disord. 2021 Sep 6;21:182. doi: 10.1186/s12902-021-00848-w (PMC8422662; doi:10.1186/s12902-021-00848-w)
Supplement: Supplementary file 1 — Additional file 1. [file 12902_2021_848_MOESM1_ESM.pdf]

Dear Sir/Madam,

Polycystic ovary syndrome (PCOS) is a well-known endocrine/metabolic disorder that affects women of reproductive age. Obesity is associated with the development of PCOS and contributes substantially to metabolic abnormalities in patients. To promote evidence-based care, patient satisfaction, and minimize long-term morbidities in women with PCOS, our team intends to conduct an online survey among obstetricians and gynecologists to know more about the practices of physicians with respect to diagnosis, evaluation, and treatment of obesity in patients with PCOS. Participation is voluntary and anonymous. Details that could identify respondents would be excluded, such as patient name, location, etc. All participants are requested to complete the survey only once. If you agree to participate in this survey, please send a message in the chat group of the China Maternal and Child Health Association, then answer all the questions. Completion of the survey and the message will be taken as your consent to participate. Thank you for your participation.

QUESTIONNAIRE

Q.1 Today's date is___________

Q.2 Province___________

Q.3 Sex

a) female

b) male

Q.4 Age (in years)

a) 18~25

b) 26~35

c) 36~45

d) 46~55

e) >55

Q.5 The level of hospital you are working in.

a) Tertiary hospital

b) Secondary hospital

c) Grade-one hospital

d) Others___________

Q.6 What type of specialty are you practicing in?

a) Reproductive endocrinologist

b) Gynaecologist

c) Obstetrician-gynaecologist

d) Obstetrician

e) Others___________

Q.7 How many patients with polycystic ovary syndrome have you seen in the last year?

a) <50

b) 50~200

c) >200

Q.8 In your estimate, what is the national prevalence of overweight/obese

patients with PCOS?

a) 0-30%

b) 31-50%

c) 51-80%

d) >80%

Q.9 What was the most common reason for consultation clinic attendance in those patients that you have diagnosed with polycystic ovary syndrome?

a) Menstrual disorders

b) Infertility

c) Hirsutism/acne

d) Obesity/IR

e) Others___________

Q.10 What was the most common diagnostic criterion that you used for the diagnosis of overweight/obesity?

a) BMI (WHO criteria)

b) BMI (Chinese criteria)

c) BMI (Asian criteria)

d) Waistline measurement

e) Others___________

Q.11 Do you ask your patients about personal or family history of diabetes, cardiovascular disease, and polycystic ovary syndrome?

(a) personal or family history of diabetes

_ Yes _ No

(b) personal or family history of cardiovascular disease

_ Yes _ No

(c) family history of polycystic ovary syndrome

_ Yes _ No

Q.12 Do you ask your patients about symptoms of anxiety, depression, and other psychological problems?

_ Yes _ No

Q.13 What were the common metabolic screening test/s that you ordered for patients with obesity and polycystic ovary syndrome?

(a) Fasting glucose ± fasting insulin

_ Yes _ No

(b) Two-hour OGTT

_ Yes _ No

(c) Two-hour OGTT + insulin test

_ Yes _ No

(d) Lipid profile

_ Yes _ No

(e) Two-hour OGTT + lipid profile

_ Yes _ No

Q.14 What were the common treatment/s that you prescribed for most of your patients with polycystic ovary syndrome without fertility requirements?

(a) Lifestyle modifications

_Yes _ No

(b) Oral contraceptives

_ Yes _ No

(c) Metformin

_ Yes _ No

(d) Thiazolidinediones

_ Yes _ No

(e) Antiandrogens

_ Yes _ No

(f) Surgery

_ Yes _ No

(g) Others____________

Q.15 What were the common treatment/s that you prescribed for most of your patients with polycystic ovary syndrome regarding fertility?

(a) Lifestyle modifications

_ Yes _ No

(b) Letrozole

_ Yes _ No

(c) Clomiphene citrate

_ Yes _ No

(d) Metformin

_ Yes _ No

(e) Thiazolidinediones

_ Yes _No

(f) Assisted reproductive technology

_ Yes _ No

(g) Surgery

_ Yes _ No

(h) Antiandrogens

_ Yes _ No

(i) Others___________

Q.16 Which method/s do you think are feasible for overweight/obese patients with polycystic ovary syndrome to lose weight?

(a) Clinical dietitian

_ Yes _ No

(b) Chinese medicine

_ Yes _ No

(c) Metformin

_ Yes _ No

(d) Orlistat

_ Yes _ No

(e) Glucagon-like peptide-1 (GLP-1) receptor agonists

_ Yes _ No

(f) Assisted reproductive technology

_ Yes _ No

(g) Bariatric surgery

_ Yes _ No

(h) Others___________

Q.17 What was the most common dosage of metformin that you used for overweight/obese patients with polycystic ovary syndrome?

a) 500 mg/day

b) 1,000 mg/day

c) 1,500 mg/day

d) ≧2,000 mg/day

e) Unclear

Thank you again for your participation.
